# Comparison between effects of pressure support and pressure-controlled ventilation on lung and diaphragmatic damage in experimental emphysema

**DOI:** 10.1186/s40635-016-0107-0

**Published:** 2016-10-19

**Authors:** Gisele de A. Padilha, Lucas F. B. Horta, Lillian Moraes, Cassia L. Braga, Milena V. Oliveira, Cíntia L. Santos, Isalira P. Ramos, Marcelo M. Morales, Vera Luiza Capelozzi, Regina C. S. Goldenberg, Marcelo Gama de Abreu, Paolo Pelosi, Pedro L. Silva, Patricia R. M. Rocco

**Affiliations:** 1 Laboratory of Pulmonary Investigation, Carlos Chagas Filho Biophysics Institute, Federal University of Rio de Janeiro, Centro de Ciências da Saúde, Av. Carlos Chagas Filho, s/n, Bloco G-014, Ilha do Fundão, Rio de Janeiro, RJ 21941-902 Brazil; 2 Laboratory of Molecular and Cellular Cardiology, Carlos Chagas Filho Biophysics Institute, Federal University of Rio de Janeiro, Rio de Janeiro, RJ Brazil; 3 National Center for Structural Biology and Bio-imaging, Federal University of Rio de Janeiro, Rio de Janeiro, RJ Brazil; 4 Laboratory of Cellular and Molecular Physiology, Carlos Chagas Filho Biophysics Institute, Federal University of Rio de Janeiro, Rio de Janeiro, Brazil; 5 Department of Pathology, School of Medicine, University of São Paulo, São Paulo, Brazil; 6 Pulmonary Engineering Group, Department of Anesthesiology and Intensive Care Therapy, University Hospital Carl Gustav Carus, Technische Universität Dresden, Dresden, Germany; 7 Department of Surgical Sciences and Integrated Diagnostics, IRCCS AOU San Martino-IST, University of Genoa, Genoa, Italy

**Keywords:** Emphysema, Pressure support ventilation, Amphiregulin, Diaphragm, Echocardiography

## Abstract

**Background:**

In patients with emphysema, invasive mechanical ventilation settings should be adjusted to minimize hyperinflation while reducing respiratory effort and providing adequate gas exchange. We evaluated the impact of pressure-controlled ventilation (PCV) and pressure support ventilation (PSV) on pulmonary and diaphragmatic damage, as well as cardiac function, in experimental emphysema.

**Methods:**

Emphysema was induced by intratracheal instillation of porcine pancreatic elastase in Wistar rats, once weekly for 4 weeks. Control animals received saline under the same protocol. Eight weeks after first instillation, control and emphysema rats were randomly assigned to PCV (*n* = 6/each) or PSV (*n* = 6/each) under protective tidal volume (6 ml/kg) for 4 h. Non-ventilated control and emphysema animals (*n* = 6/group) were used to characterize the model and for molecular biology analysis. Cardiorespiratory function, lung histology, diaphragm ultrastructure alterations, extracellular matrix organization, diaphragmatic proteolysis, and biological markers associated with pulmonary inflammation, alveolar stretch, and epithelial and endothelial cell damage were assessed.

**Results:**

Emphysema animals exhibited cardiorespiratory changes that resemble human emphysema, such as increased areas of lung hyperinflation, pulmonary amphiregulin expression, and diaphragmatic injury. In emphysema animals, PSV compared to PCV yielded: no changes in gas exchange; decreased mean transpulmonary pressure (Pmean,L), ratio between inspiratory and total time (Ti/Ttot), lung hyperinflation, and amphiregulin expression in lung; increased ratio of pulmonary artery acceleration time to pulmonary artery ejection time, suggesting reduced right ventricular afterload; and increased ultrastructural damage to the diaphragm. Amphiregulin correlated with Pmean,L (*r* = 0.99, *p* < 0.0001) and hyperinflation (*r* = 0.70, *p* = 0.043), whereas Ti/Ttot correlated with hyperinflation (*r* = 0.81, *p* = 0.002) and Pmean,L (*r* = 0.60, *p* = 0.04).

**Conclusions:**

In the model of elastase-induced emphysema used herein, PSV reduced lung damage and improved cardiac function when compared to PCV, but worsened diaphragmatic injury.

**Electronic supplementary material:**

The online version of this article (doi:10.1186/s40635-016-0107-0) contains supplementary material, which is available to authorized users.

## Background

Emphysema is characterized by abnormal permanent enlargement of airspaces distal to the terminal bronchiole accompanied by destruction of their walls, resulting in decreased elastic recoil, hyperinflation, and air trapping [1]. Additionally, patients with emphysema experience cardiovascular impairment [2] and diaphragmatic dysfunction [3] associated with muscle proteolysis [4].

Noninvasive positive-pressure ventilation has been considered the first-line treatment of choice for exacerbations of emphysema but is not always suitable in more severe cases [5], which often require invasive mechanical ventilation. When providing ventilatory support to patients with emphysema, the main goals are to improve gas exchange and avoid or prevent further aggravation of hyperinflation, cardiovascular dysfunction, and diaphragmatic atrophy. Controlled mechanical ventilation has been shown to induce muscle atrophy and alter the contractile properties of the diaphragm in healthy lungs in experimental [6] and clinical [7] settings, as well as in critically ill patients [8]. Pressure support ventilation requires less sedation, no paralysis, and has been associated with improved gas exchange and lung mechanics (due to decreased time-constant inhomogeneity), as well as with less hemodynamic deterioration and diaphragmatic damage, in experimental [6, 9, 10] and clinical [11] reports. To date, however, no study has evaluated the impact of pressure support ventilation (PSV) and pressure-controlled ventilation (PCV) on lung damage, diaphragmatic injury, and cardiovascular function in experimental emphysema.

In the present study, we compared the effects of PSV vs. PCV on lung and cardiac function, pulmonary histology, ultrastructural changes in diaphragm tissue, and biological markers associated with pulmonary inflammation, alveolar stretch, epithelial mechanotransduction, endothelial cell damage, extracellular matrix organization, and diaphragmatic proteolysis in an animal model of elastase-induced emphysema. We hypothesized that PSV would improve cardiorespiratory function and reduce pulmonary and diaphragmatic damage in experimental emphysema.

## Methods

### Animal preparation and experimental protocol

This study was approved by the Research Ethics Committee of the Federal University of Rio de Janeiro Health Sciences Center (CEUA 019). All animals received humane care in compliance with the Principles of Laboratory Animal Care formulated by the National Society for Medical Research and the U.S. National Academy of Sciences.

#### Guide for the care and use of laboratory animals

Thirty-six male Wistar rats (weighing 256 ± 15 g) were kept in controlled-temperature conditions (23 °C) and maintained on a 12:12 h light–dark cycle with free access to water and food. Animals were randomly divided into two groups. In the emphysema group, rats were administered porcine pancreatic elastase (2 IU suspended in saline solution to a total volume of 100 μl, Sigma Chemical Co., St. Louis, MO, USA) intratracheally (it), once weekly for 4 weeks, whereas control animals received saline solution alone (100 μl) under the same protocol. Before each intratracheal instillation, rats were premedicated with intraperitoneal (ip) diazepam (10 mg/kg, Compaz®, Cristália, Itapira, SP, Brazil) and anesthetized with 1.5–2.0 % isoflurane (Cristália, Itapira, SP, Brazil) by mask.

**Fig. 1:**
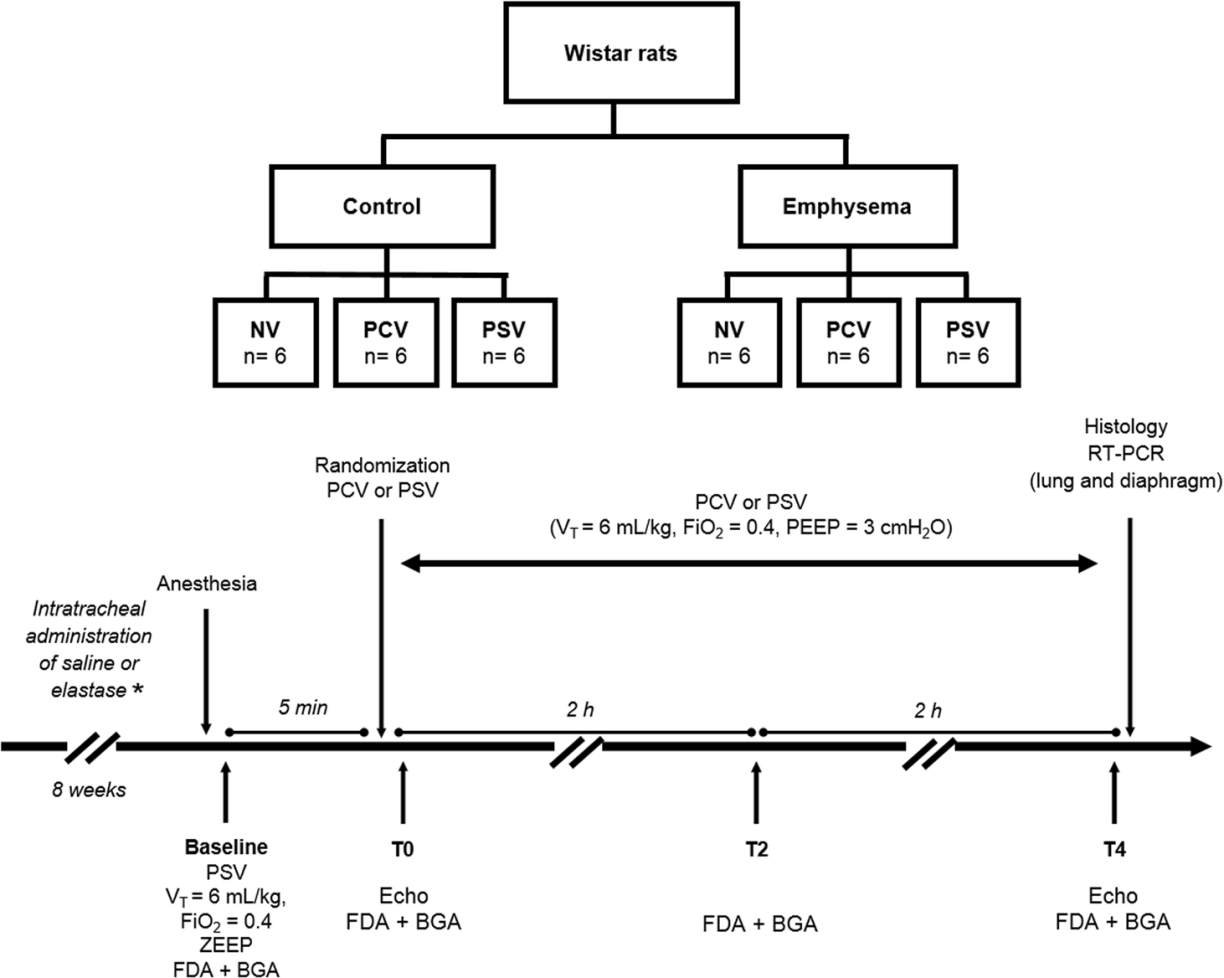
Schematic flowchart and timeline of the experimental protocol. *NV* non-ventilated, *PCV* pressure-controlled ventilation, *PSV* pressure support ventilation, *V*_*T*_ tidal volume, *FiO*_*2*_ fraction of inspired oxygen, *ZEEP* zero end-expiratory pressure, *FDA* functional data acquisition, *BGA* blood gas-analysis, *Echo* echocardiography, *PEEP* positive end-expiratory pressure, *RT-PCR* real-time reverse transcription polymerase chain reaction. T0—immediately after randomization; T2 and T4—2 and 4 h of mechanical ventilation after randomization, respectively

Eight weeks after the first instillation (Fig. 1), rats (weighing 398 ± 23 g) were sedated with diazepam (10 mg/kg ip, Compaz®, Cristália, Itapira, SP, Brazil) and anesthetized with ketamine (100 mg/kg ip, Ketamin-S+®, Cristália, Itapira, SP, Brazil) and midazolam (2 mg/kg ip, Dormicum, União Química, São Paulo, SP, Brazil). The tail vein was cannulated (Jelco 24G, BD, New Jersey, USA) for continuous infusion of 50 mg/kg/h ketamine, 2 mg/kg/h midazolam, and 7 ml/kg/h Ringer’s lactate (B. Braun, Rio de Janeiro, Brazil) during mechanical ventilation. The adequacy of anesthesia was assessed by the response to a nociceptive stimulus before surgery.

Anesthetized animals were placed in the dorsal recumbent position and tracheotomized via a midline neck incision after subcutaneous injection of lidocaine (Xylestesin® 2 %, Cristália, Itapira, SP, Brazil). The right internal carotid artery was cannulated (18G, Arrow International, USA) for blood sampling and mean arterial pressure (MAP) measurement.

Heart rate (HR), MAP, and rectal temperature were continuously recorded (Networked Multiparameter Veterinary Monitor LifeWindow 6000V, Digicare Animal Health, Florida, USA). Body temperature was maintained at 37.5 ± 1 °C using a heating bed. Gelafundin® (B. Braun, São Gonçalo, RJ, Brazil) was administered intravenously in 0.5-ml increments to keep MAP ≥ 70 mmHg.

Once animals were hemodynamically stable, they were mechanically ventilated (Servo-i, MAQUET, Solna, Sweden) in PSV mode for 5 min, with a tidal volume (*V*_T_) of 6 ml/kg, zero end-expiratory pressure (ZEEP), and fraction of inspired oxygen (FiO_2_) set to 0.4, to evaluate whether the degree of lung damage was similar in emphysema groups. Arterial blood (300 μl) was drawn into a heparinized syringe for the measurement of arterial oxygen partial pressure (PaO_2_), arterial carbon dioxide partial pressure (PaCO_2_), arterial pH (pHa), and bicarbonate (Radiometer ABL80 FLEX, Copenhagen NV, Denmark). At this time (baseline), data on MAP, rectal temperature, and respiratory parameters were collected for functional data analysis (FDA). Following this step, control and emphysema animals were randomly assigned by the sealed-envelope method to receive mechanical ventilation in PCV or PSV mode (*n* = 6/each). During PCV, animals were paralyzed by intravenous administration of pancuronium bromide (2 mg/kg, Cristália, Itapira, SP, Brazil). In PCV and PSV, driving pressure was adjusted to achieve a *V*_T_ of 6 ml/kg, and positive end-expiratory pressure (PEEP) was set at 3 cmH_2_O and FiO_2_ = 0.4. In addition, in PCV, the respiratory rate (RR) was controlled to keep minute ventilation constant at 160 ml/min. Six animals from each group (control and emphysema) were not ventilated (NV) and used as controls for experimental emphysema characterization and molecular biology analysis. Blood gases and respiratory parameters were analyzed immediately after randomization (time point 0, T0) and after 2 h (T2) and 4 h (T4) of mechanical ventilation, whereas echocardiography was performed at T0 and T4. At the end of the experiment, heparin (1000 IU) was injected into the tail vein, a laparotomy was performed, and animals were killed by intravenous injection of sodium thiopental (50 mg/kg, Cristália, Itapira, SP, Brazil). The left and right lungs were extracted at an airway pressure equivalent to PEEP for histological and molecular biology analysis, respectively. The lungs and diaphragm of NV animals were extracted for lung histology and molecular biology analysis. Schematic flowcharts of study design and a timeline representation of the protocol are shown in Fig. 1.

### Echocardiography

Animals were placed in the dorsal recumbent position and the precordial region was shaved. Transthoracic echocardiography was performed by an expert (IPR) blinded to group allocation, using a 7.5-MHz probe (Esaote model, CarisPlus, Firenze, Italy). Images were obtained from the parasternal views. The left ventricular ejection fraction and fractional shortening were calculated in one-dimensional mode analysis of the left ventricle guided by the parasternal short-axis view. Pulsed-wave Doppler was used to measure pulmonary artery acceleration time (PAT) and pulmonary artery ejection time (PET), and the PAT/PET ratio was used as an indirect index of pulmonary arterial hypertension. Measurements were obtained in accordance with American Society of Echocardiography Guidelines [12, 13].

### Respiratory data acquisition and processing

A pneumotachograph (internal diameter = 1.5 mm, length = 4.2 cm, distance between side ports = 2.1 cm) was connected to the tracheal cannula for airflow (V’) measurements. The pressure gradient across the pneumotachograph was determined using a SCIREQ differential pressure transducer (UT-PDP-300, SCIREQ, Montreal, Canada). *V*_T_ was calculated by digital integration of the flow signal. Tracheal pressure (Paw) was measured with a SCIREQ differential pressure transducer (UT-PDP-75, SCIREQ, Montreal, QC, Canada). Changes in esophageal pressure (Pes), which reflect chest wall pressure, were measured with a 30-cm-long water-filled catheter (PE205) with side holes at the tip connected to a differential pressure transducer (UT-PL-400, SCIREQ, Montreal, Canada). The catheter was passed into the stomach and then slowly returned into the esophagus; its proper positioning was assessed using the “occlusion test” [14]. Transpulmonary pressure (P,L) was calculated during inspiration and expiration as the difference between tracheal and esophageal pressures. Mean (Pmean,L) and peak transpulmonary pressures (Ppeak,L) were calculated. The respiratory rate (RR) was calculated from Pes swings as the frequency per minute of each type of breathing cycle. The ratio between inspiratory and total time (Ti/Ttot) was calculated, as well as the coefficients of variation of *V*_T_, RR, and Ti/Ttot. Moreover, the esophageal pressure generated 100 ms after onset of inspiratory effort (*P*_*0*.1_) and the pressure–time product (PTP) per minute (PTP/min) (integral of ΔPes over time) were calculated.

Airflow and tracheal and esophageal pressures were continuously recorded throughout the experiments with a computer running software written in LabVIEW® (National Instruments; Austin, Texas, USA) (Additional file 1: Figure S1). All signals were filtered (200 Hz), amplified by a 4-channel conditioner (SC-24, SCIREQ, Montreal, Quebec, Canada), and sampled at 200 Hz with a 12-bit analogue-to-digital converter (National Instruments; Austin, Texas, USA). All mechanical data were computed offline by a routine written in MATLAB (Version R2007a; The Mathworks Inc, Natik, Massachusetts, USA).

### Lung histology

Morphometric analysis was performed in excised lungs at end-expiration with a PEEP of 3 cmH_2_O. Immediately after excision, the left lung was flash-frozen by immersion in liquid nitrogen, fixed with Carnoy’s solution, and embedded in paraffin. Slices (4 μm thick) were mounted and stained with hematoxylin–eosin. Morphometric analysis was done by viewing through an integrating eyepiece with a coherent system made of a 100-point grid consisting of 50 lines of known length, coupled to a conventional light microscope (Axioplan, Zeiss, Oberkochen, Germany). The volume fraction of collapsed pulmonary areas and the fraction of the lung occupied by large-volume gas-exchanging air spaces (hyperinflated structures with a morphology distinct from that of alveoli and wider than 120 μm) were determined by the point-counting technique, at a magnification of ×200, across ten random, non-coincident microscopic fields [15]. Briefly, points falling on collapsed pulmonary or hyperinflated areas were counted and divided by the total number of points in each microscopic field. Lung tissue distortion was assessed by measuring the mean linear intercept between alveolar walls (Lm) at a magnification of ×400 [16]. Lm is an estimate of the average difference between gas exchange surfaces. The investigators (LFHB, CLB) were unaware of the origin of the examined material.

### Transmission electron microscopy of diaphragm tissue

Three slices (each 2 × 2 × 2 mm) were cut from three different segments of the diaphragm and fixed in 2.5 % glutaraldehyde and phosphate buffer 0.1 M (pH = 7.4) for electron microscopy analysis (JEOL 1010 Transmission Electron Microscope; Japan Electron Optics Laboratory Co, Tokyo, Japan). The following parameters were observed qualitatively: (1) fibrillar disarrangement, (2) thickened Z-line, (3) smooth cell proliferation, (4) abnormal mitochondria, and (5) enlarged endoplasmic reticulum [17]. To assess pathological findings, a five-point, semi-quantitative, severity-based scoring system was used as follows: 0 = normal diaphragm, 1 = changes in 1 to 25 % of examined tissue, 2 = changes in 26 to 50 % of examined tissue, 3 = changes in 51 to 75 % of examined tissue, and 4 = changes in 76 to 100 % of examined tissue. Scores were calculated as the product of severity and extent of each feature, ranging from 0 to 16. This analysis was performed by a pathologist (VLC) blinded to group allocation.

### Molecular biology analysis of lung and diaphragm tissue

Quantitative real-time reverse transcription polymerase chain reaction (RT-PCR) was performed to measure biological markers associated with cell mechanical stress (amphiregulin), inflammation (cytokine-induced neutrophil chemoattractant (CINC-1)), epithelial cell mechanotransduction (surfactant protein (SP)-D), endothelial cell damage (vascular endothelial growth factor (VEGF), vascular cell adhesion molecule (VCAM)-1, and angiopoietin (ANG)-2), extracellular matrix organization (lysyl oxidase-like (LOXL)1), and fibrogenesis (type III procollagen (PCIII)) in the lung, as well as markers of muscle proteolysis (muscle atrophy F-box (MAFbx) and muscle RING finger (MuRF)-1). Central slices were cut from the right lung and diaphragm, collected in cryotubes, flash-frozen in liquid nitrogen, and stored at −80 °C. Total RNA was extracted from frozen tissues using the RNeasy Plus Mini Kit (Qiagen, Hilden, Germany) for the lungs and RNeasy Fibrous tissue Mini Kit (Qiagen, Hilden, Germany) for the diaphragm, following the manufacturer’s recommendations. The RNA concentration was measured by spectrophotometry in a Nanodrop ND-1000 system. First-strand cDNA was synthesized from total RNA using a Quantitec reverse transcription kit (Qiagen, Hilden, Germany). The primers used are described in the Supplementary Material (Additional file 2: Table S1). Relative messenger RNA (mRNA) levels were measured with a SYBR green detection system using real-time PCR (ABI 7500; Applied Biosystems, Foster City, CA, USA). For each sample measured in triplicate, the gene expression was normalized to that of a housekeeping gene (acidic ribosomal phosphoprotein P0, *36B4*) [18] and expressed as fold change relative to non-ventilated control and emphysema animals, using the 2^−ΔΔ^Ct method, where ΔCt = Ct (reference gene) minus Ct (target gene). This is a suitable method to analyze relative changes in gene expression from real-time quantitative PCR experiments [19].

### Statistical analysis

The number of animals per group was based on a previous study [17]. A sample size of six animals per group (providing for one animal as dropout) would provide the appropriate power (1-β = 0.8) to identify significant (*α* = 0.05) differences in mean transpulmonary pressure obtained after PSV and PCV, taking into account an effect size *d* = 1.6, a two-sided test, and a sample size ratio = 1 (G*Power 3.1.9.2, University of Düsseldorf, Germany).

Data were tested for normality using the Kolmogorov-Smirnov test with Lilliefors’ correction, while the Levene median test was used to evaluate the homogeneity of variances. If both conditions were satisfied, two-way ANOVA followed by Tukey’s test was used. To compare the time course of respiratory parameters and arterial blood gases, one-way repeated-measures ANOVA followed by Bonferroni’s test was used. A *t* test with Bonferroni correction was used to compare PCV and PSV in control and emphysema groups. For nonparametric data, the Kruskal-Wallis test followed by Dunn’s post hoc test was used. Parametric data were expressed as mean ± standard deviation (SD), and nonparametric data, as median (interquartile range). Spearman’s correlations were used. All tests were performed using the GraphPad Prism v6.01 statistical software package (GraphPad Software, La Jolla, California, USA). Significance was accepted at *p* < 0.05.

## Results

### Characterization of elastase-induced emphysema model

Emphysema animals exhibited increased areas of lung hyperinflation, mean linear intercept, amphiregulin mRNA expression in lung tissue, MAFbx expression in diaphragm, and a reduced PAT/PET ratio compared to control animals (Additional file 3: Table S2).

### Effects of pressure support and pressure-controlled ventilation in control animals

At baseline, before randomization, all control animals were evaluated during PSV to confirm the absence of any significant differences between PSV and PCV regarding arterial blood gases and respiratory parameters (Additional file 4: Table S3).

**Fig. 2:**
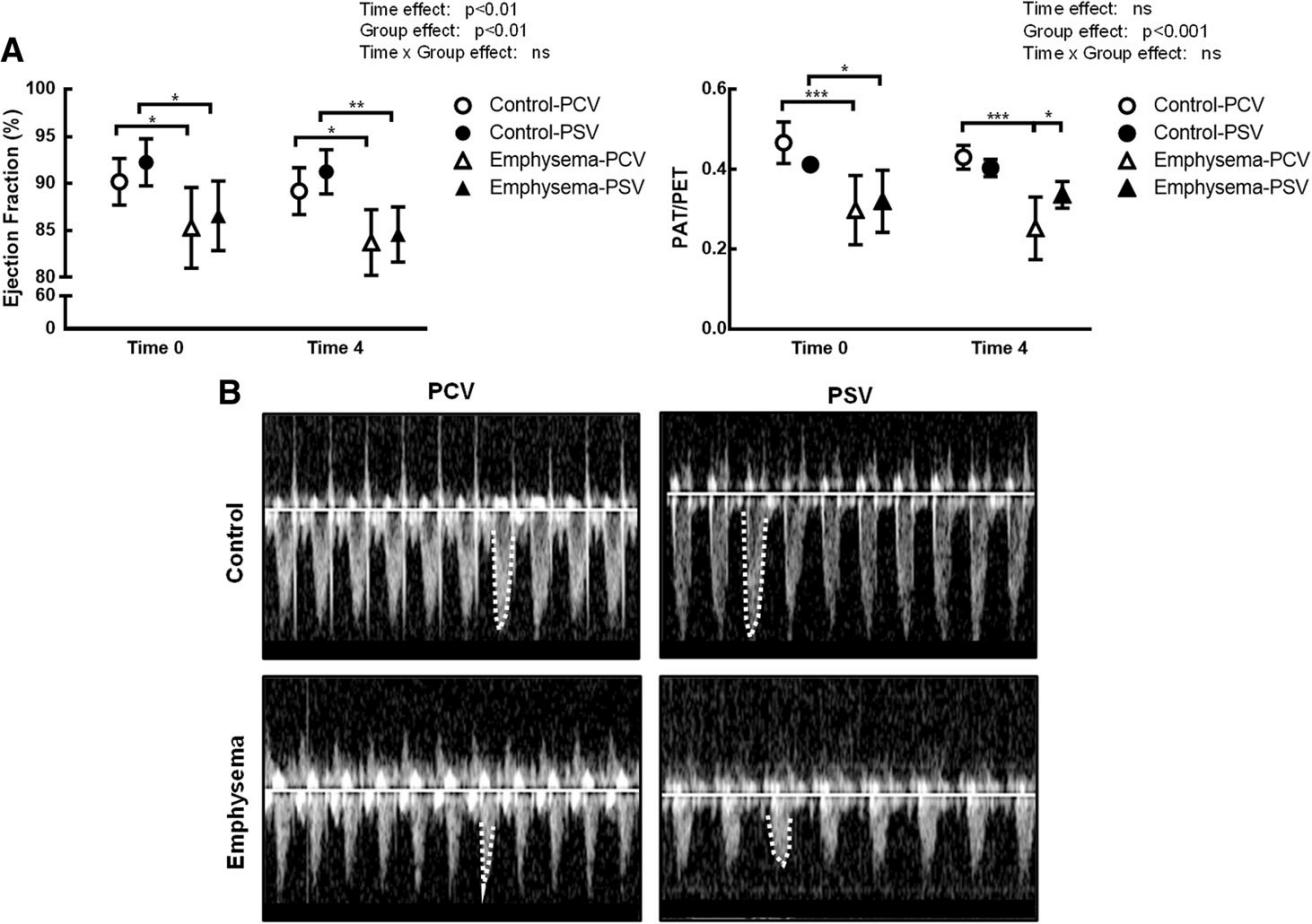
Echocardiographic analysis. **a** Ejection fraction (EF) and ratio of pulmonary artery acceleration time (PAT) to pulmonary artery ejection time (PET). The PAT/PET ratio was used as an indirect index of pulmonary arterial hypertension. **b** Representative images of pulmonary blood flow at T4. Note the spiculated pattern of the pulmonary artery flow curve in PCV and bell-shaped curve in PSV. *PCV* pressure-controlled ventilation, *PSV* pressure support ventilation. Symbols are means ± SD of six animals. *p < 0.05, **p < 0.01, ***p < 0.001

**Fig. 3:**
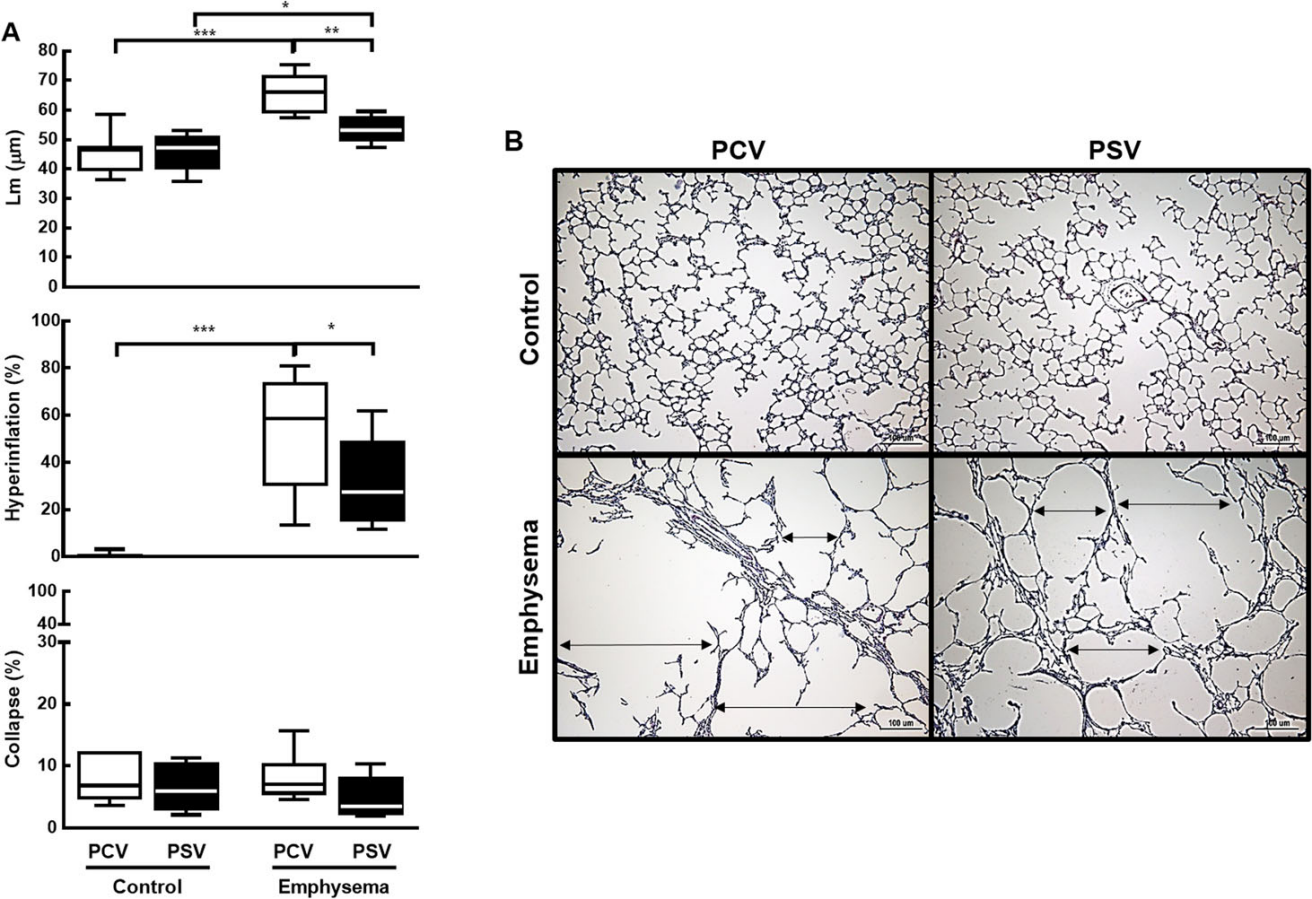
Lung morphometry. **a** Mean linear intercept (Lm), fraction area of hyperinflated and collapsed alveoli. **b** Representative photomicrographs of lung parenchyma stained with hematoxylin–eosin. Note alveolar space enlargement in the emphysema group (*arrows*). *PCV* pressure-controlled ventilation, *PSV* pressure support ventilation. *Boxes* show the interquartile (25–75 %) range, *whiskers* encompass the range (minimum–maximum), and *horizontal lines* represent the median in six animals/group. *p < 0.05, **p < 0.01, ***p < 0.001

**Fig. 4:**
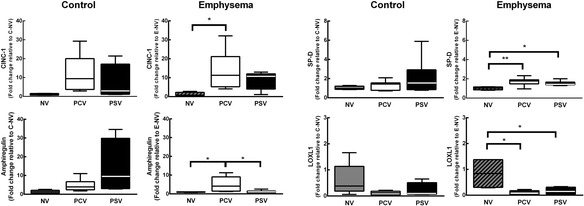
Expression of biological markers in lung tissue. Real-time polymerase chain reaction analysis of cytokine-induced neutrophil chemoattractant-1 (CINC-1), amphiregulin, surfactant protein (SP)-D, and lysyl oxidase (LOX)-2. Relative gene expression was calculated as a ratio of the average gene expression levels compared with the reference gene (*36B4*) and expressed as fold changes relative to NV (non-ventilated) animals in the control and emphysema groups. *PCV* pressure-controlled ventilation, *PSV* pressure support ventilation. *Boxes* show the interquartile (25–75 %) range, *whiskers* encompass the range (minimum–maximum), and *horizontal lines* represent the median in six animals/group. *p < 0.05, **p < 0.01

**Fig. 5:**
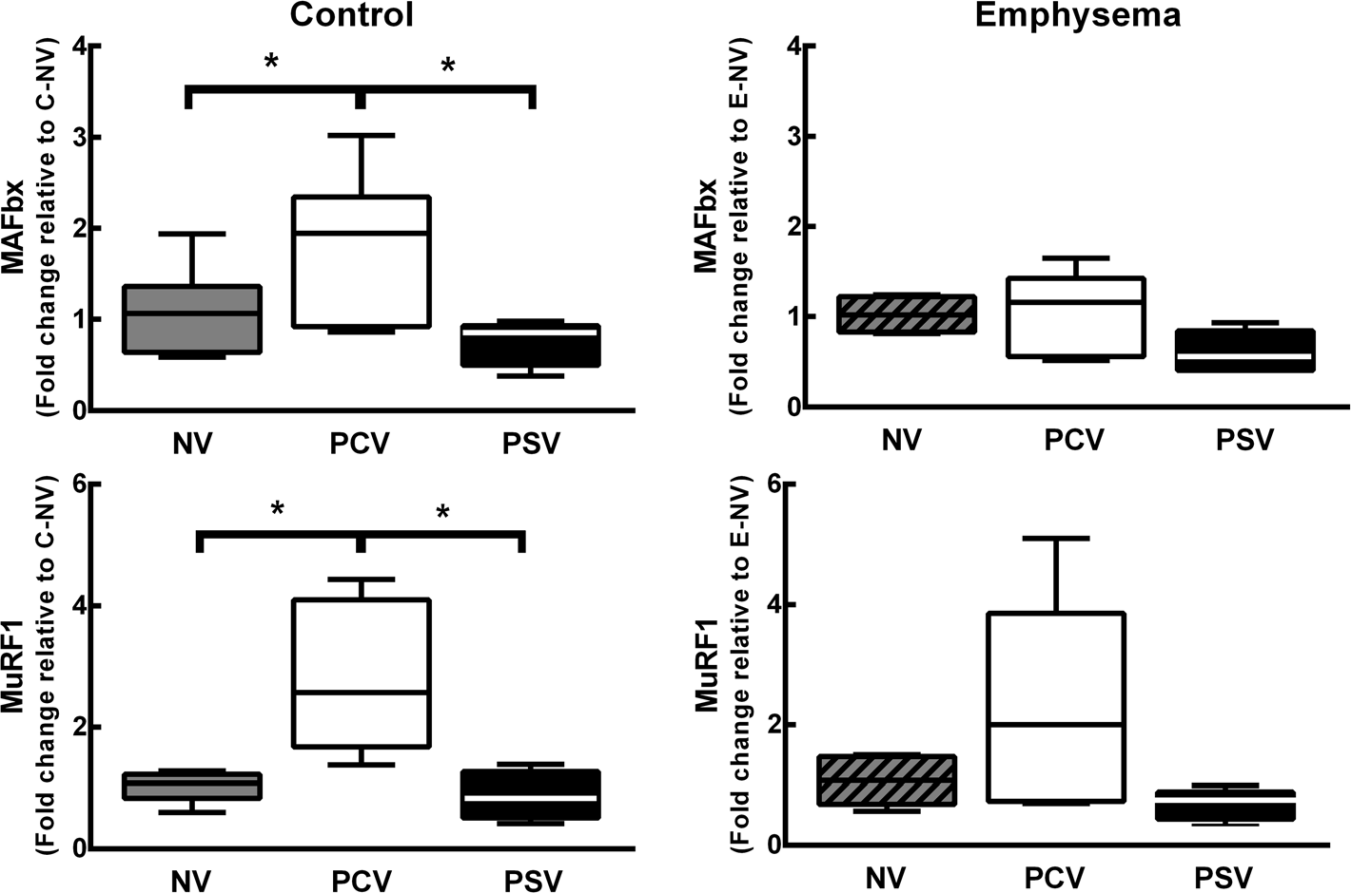
Expression of biological markers in diaphragm tissue. Real-time polymerase chain reaction analysis of biological markers associated with muscle proteolysis: muscle atrophy F-Box (MAFbx) and muscle RING finger-1 (MuRF1). Relative gene expression was calculated as a ratio of the average gene expression levels compared with the reference gene (*36B4*) and expressed as fold change relative to NV (non-ventilated) animals in the control and emphysema groups. *PCV* pressure-controlled ventilation, *PSV* pressure support ventilation. *Boxes* show the interquartile (25–75 %) range, *whiskers* encompass the range (minimum–maximum), and *horizontal lines* represent the median in six animals/group.*p < 0.05, **p < 0.01

**Fig. 6:**
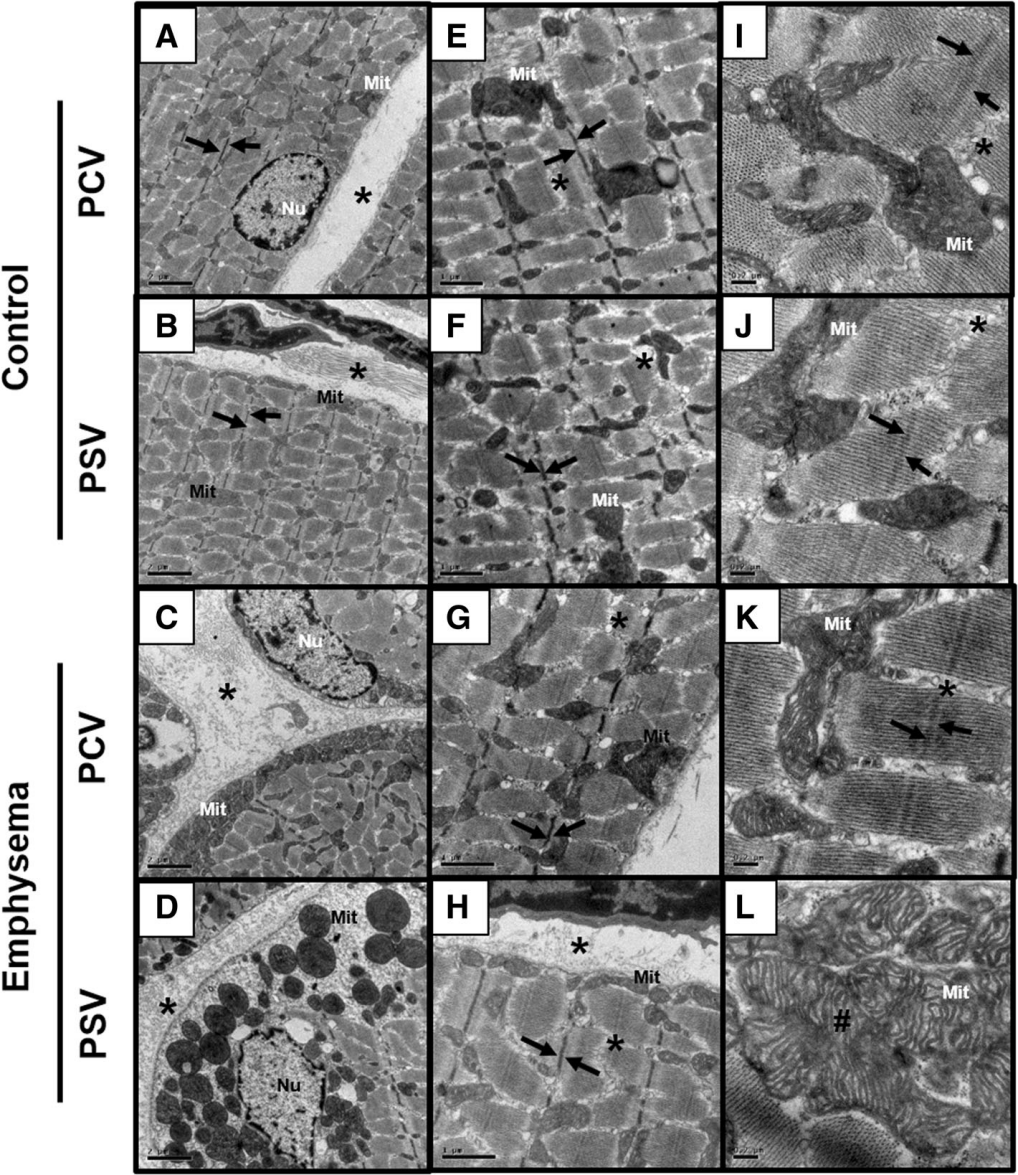
Transmission electron microscopy of diaphragm tissue. Electron microscopy of the diaphragm muscle in control and emphysema animals ventilated with PCV and PSV, visualized under different magnifications. At low magnification, control animals exhibited muscle fiber composed of myofibrils separated in fascicles by the sarcomeres (*arrows*), containing a peripheral nucleus with regular chromatin and clusters of mitochondria arranged in the periphery or middle of the sarcoplasm (**a**, **b**, **e**, **f**). At high magnification, mitochondrial cristae are lightly irregular and distorted in control animals under PCV (**i**) and PSV (**j**). Prominent edema (*asterisk*) and irregularity of the mitochondrial cristae (hashtag) can be observed in emphysema animals under PSV (**l**) compared to emphysema animals under PCV (**c**,**g**) PSV (**d**,**h**,**l**) compared to emphysema animals under PCV (**k**). *PCV* pressure-controlled ventilation, *PSV* pressure support ventilation, *Mit* mitochondria, *Nu* nucleus

After randomization, *V*_T_, Ti/Ttot, and PPeak,L remained unaltered in PSV compared to PCV animals at T4 (Table 1). Between-group differences were observed in Ti/Ttot at T2 and Ppeak,L at T0. Compared to PCV, PSV increased the coefficient of variation of *V*_T_, RR, and Ti/Ttot, but decreased RR and Pmean,L. PTP/min and *P*_0.1_ did not change during the time course of mechanical ventilation (Table 1). MAP (Additional file 5: Table S4), arterial blood gases (Additional file 6: Table S5), ejection fraction, and PAT/PET (Fig. 2) did not differ between PCV and PSV or change during the time course of mechanical ventilation. MAP remained stable and >70 mmHg throughout the experiments (Additional file 5: Table S4), and the volume of fluids infused was similar in both groups (PCV, 8.1 ± 2.4 ml; PSV, 11.1 ± 5.7 ml). Lung morphometry (Fig. 3) and expression of CINC-1, amphiregulin, SP-D, LOXL1 (Fig. 4), VEGF, ANG-2, PCIII, and VCAM-1 (Additional file 7: Table S6) did not differ significantly between PCV and PSV. Even though expression of MAFbx and MuRF1 was higher in PCV than PSV (Fig. 5), no significant changes in diaphragm ultrastructure were found (Table 2, Fig. 6).

**Table 1:** Respiratory parameters

Parameter	Group		T0	T2	T4	Time effect	Group effect	Interaction
*V*_T_ (mL.kg^−1^)	Control	PCV	5.9 ± 0.4	5.7 ± 0.5	5.7 ± 0.5	ns	ns	ns
		PSV	6.5 ± 0.9	6.6 ± 1.0	6.6 ± 0.7			
	Emphysema	PCV	6.0 ± 1.0	5.9 ± 0.6	6.1 ± 0.5			
		PSV	6.1 ± 1.1	6.3 ± 1.1	6.3 ± 1.1			
RR (breaths.min^−1^)	Control	PCV	62.1 ± 4.7	62.1 ± 4.6	62.2 ± 4.5	*p* < 0.05	*p* < 0.001	*p* < 0.05
		PSV	39.8 ± 4.9*	44.9 ± 5.6*	54.6 ± 12.7			
	Emphysema	PCV	59.5 ± 4.0	60.8 ± 2.9	59.8 ± 3.5			
		PSV	56.7 ± 10.8**	52.9 ± 7.8	62.0 ± 11.0			
Ti/Ttot (s)	Control	PCV	0.35 ± 0.00	0.35 ± 0.01	0.35 ± 0.00	ns	*p* < 0.05	ns
		PSV	0.31 ± 0.10	0.31 ± 0.07	0.32 ± 0.05			
	Emphysema	PCV	0.35 ± 0.00	0.35 ± 0.00	0.35 ± 0.01			
		PSV	0.29 ± 0.05	0.27 ± 0.02***	0.31 ± 0.06			
Ppeak,L (cmH_2_O)	Control	PCV	10.8 ± 1.9	11.2 ± 1.5	12.1 ± 1.4	ns	*p* < 0.01	*p* < 0.05
		PSV	12.6 ± 1.8	13.0 ± 2.2	12.3 ± 3.1			
	Emphysema	PCV	11.3 ± 1.5	12.2 ± 1.5	13.5 ± 1.3			
		PSV	15.9 ± 2.0**^,^ ***	15.7 ± 1.7	14.3 ± 2.3			
Pmean,L (cmH_2_O)	Control	PCV	5.8 ± 0.7	5.8 ± 06	6.3 ± 0.4	*p* < 0.05	*p* < 0.001	ns
		PSV	4.8 ± 0.6	5.1 ± 0.6	5.0 ± 0.7*			
	Emphysema	PCV	5.9 ± 0.6	6.2 ± 0.6	6.7 ± 0.4			
		PSV	5.8 ± 0.6**	5.5 ± 0.4	5.3 ± 0.5***			
PTP/min (cmH_2_O.s.min^−1^)	Control	PCV	–	–	–	ns	ns	ns
		PSV	13.9 ± 13.3	14.8 ± 12.5	17.5 ± 17.8			
	Emphysema	PCV	–	–	–			
		PSV	18.5 ± 15.0	14.9 ± 13.5	30.4 ± 14.3			
*P*_0.1_ (cmH_2_O)	Control	PCV	–	–	–	ns	ns	ns
		PSV	−1.03 ± 0.93	−1.25 ± 0.90	−1.48 ± 0.83			
	Emphysema	PCV	–	–	–			
		PSV	−1.47 ± 0.97	−1.47 ± 1.20	−2.49 ± 1.41			
CV of *V*_T_ (%)	Control	PCV	0.8 ± 0.6	2.1 ± 1.8	2.7 ± 3.7	ns	*p* < 0.0001	ns
		PSV	6.4 ± 3.9	8.0 ± 4.6*	9.5 ± 3.5*			
	Emphysema	PCV	1.9 ± 1.5	2.2 ± 1.5	2.4 ± 1.6			
		PSV	6.0 ± 3.1	7.2 ± 4.5	7.7 ± 7.3			
CV of RR (%)	Control	PCV	0.7 ± 0.0	1.3 ± 0.0	2.4 ± 0.0	ns	*p* < 0.001	ns
		PSV	49.5 ± 0.3*	48.0 ± 0.4*	34.7 ± 0.2*			
	Emphysema	PCV	0.6 ± 0.0	0.6 ± 0.0	1.3 ± 0.0			
		PSV	21.0 ± 0.2**^,^ ***	20.9 ± 0.1**^,^ ***	18.4 ± 0.1**^,^ ***			
CV of Ti/Ttot (%)	Control	PCV	1.3 ± 0.5	1.6 ± 0.7	1.8 ± 0.7	ns	*p* < 0.0001	ns
		PSV	30.0 ± 7.7*	27.6 ± 11.3*	24.1 ± 8.7*			
	Emphysema	PCV	0.9 ± 0.3	1.3 ± 0.6	1.6 ± 0.9			
		PSV	17.0 ± 9.7**^,^ ***	20.2 ± 10.2***	18.8 ± 7.1***			

Values are means ± SD of 6 animals

*PCV* pressure-controlled ventilation, *PSV* pressure support ventilation, *V*_*T*_ tidal volume, *CV of V*_*T*_ coefficient of variation of tidal volume, *RR* respiratory rate, *CV of RR* coefficient of variation of respiratory rate, *Ti/Ttot* inspiratory time divided by total respiratory cycle time, *CV of Ti/Ttot* coefficient of variation on inspiratory time divided by total respiratory cycle time, *Ppeak,L* transpulmonary peak pressure, *Pmean,L* transpulmonary mean pressure, *PTP/min* pressure–time product per minute, *P*_*0.1*_ esophageal pressure generated 100 ms after onset of inspiratory effort

*Significantly different from control-PCV (*p* < 0.05); **significantly different from Control-PSV (p < 0.05); ***significantly different from emphysema-PCV (*p* < 0.05)

**Table 2:** Findings on transmission electron microscopy of diaphragm tissue

Group		Fibrillar disarrangement	Thickened Z-line	Smooth cell proliferation	Abnormal mitochondria	Enlarged ER
Control	PCV	1.0 (1.0–1.5)	1.0 (1.0–2.0)	2.0 (1.0–3.0)	1.0 (1.0–1.0)	2.0 (1.0–2.0)
	PSV	2.0 (1.5–3.0)	2.0 (1.5–2.0)	1.0 (1.0–2.0)	1.0 (1.0–2.0)	1.0 (1.0–2.5)
Emphysema	PCV	6.0 (6.0–7.5)*	6.0 (4.0–6.0)	4.0 (2.0–4.0)	6.0 (4.0–7.5)*	4.0 (2.0–6.0)
	PSV	12.0 (12.0–16.0)**^,^ ***	6.0 (5.0–6.0)**	2.0 (1.5–4.0)	16.0 (16.0–16.0)**^,^ ***	16.0 (16.0–16.0)**^,^ ***

Values are median (interquartile range) of five animals in each group

*PCV* pressure-controlled ventilation, *PSV* pressure support ventilation, *ER* endoplasmic reticulum

*Significantly different from control-PCV (*p* < 0.05); **significantly different from control-PSV (*p* < 0.05); ***significantly different from emphysema-PCV (*p* < 0.05)

### Effects of pressure support and pressure-controlled ventilation in emphysema animals

At baseline, before randomization, all emphysema animals were evaluated during PSV to confirm the absence of significant differences in arterial blood gases and respiratory parameters between PSV and PCV (Additional file 4: Table S3).

After randomization, *V*_T_ did not differ, Ti/Ttot decreased at T2, and PPeak,L was increased only at T0 (immediately after randomization) in PSV compared to PCV (Table 1). The coefficients of variation of RR and Ti/Ttot increased, whereas Pmean,L decreased in PSV compared to PCV (Table 1). MAP (which remained stable and >70 mmHg; Additional file 4: Table S3) and arterial blood gases (Additional file 5: Table S4) did not differ between PCV and PSV and did not change during mechanical ventilation, while PAT/PET was increased in PSV compared to PCV at the end of mechanical ventilation (Fig. 2a). In addition, PCV animals showed a pulmonary artery flow curve with a spiculated pattern, while PSV animals presented a bell-shaped curve (Fig. 2b). The amount of fluids infused did not differ between groups (PCV, 10.7 ± 4.7 ml; PSV, 9.4 ± 1.6 ml). Oxygenation improved during the time course of mechanical ventilation in both PCV and PSV (Additional file 6: Table S5). Mean linear intercept and hyperinflation (Fig. 3), as well as amphiregulin expression (Fig. 4), were more reduced in PSV compared to PCV. Expressions of CINC-1, SP-D, LOXL1 (Fig. 4), VEGF, ANG-2, PCIII, and VCAM-1 (Additional file 6: Table S5) did not differ significantly between groups. PSV resulted in greater diaphragmatic damage—characterized by increased fibrillary disarrangement, abnormal mitochondria, and endoplasmic reticulum enlargement—compared to PCV (Table 2 and Fig. 6), without significant differences in MAFbx or MuRF1 expression (Fig. 5).

**Fig. 7:**
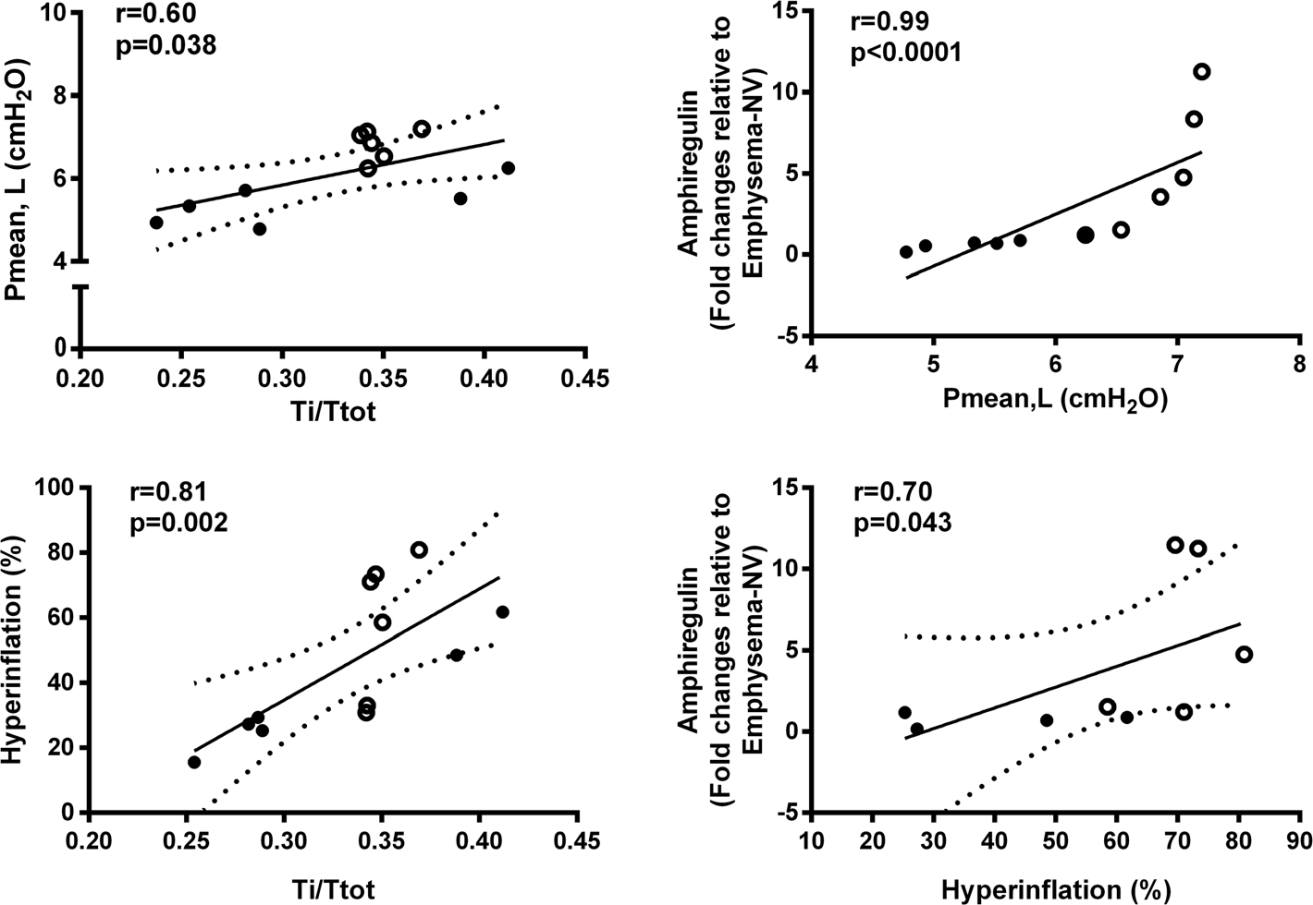
Spearman correlation between mean transpulmonary pressure (Pmean,L) and ratio between inspiratory and total time (Ti/Ttot) and amphiregulin (*upper panels*). Correlation between hyperinflation with Ti/Ttot and amphiregulin (*lower panels*). The *r* value represents the correlation coefficient, and *p*, the respective *p* value. Statistical significance was accepted at *p* < 0.05. *Black circles*: emphysema animals ventilated with PSV. *White circles*: emphysema animals ventilated with PCV

Amphiregulin correlated with Pmean,L (*r* = 0.99, *p* < 0.0001) and hyperinflation (*r* = 0.70, *p* = 0.043), whereas Ti/Ttot correlated with hyperinflation (*r* = 0.81, *p* = 0.002) and Pmean,L (*r* = 0.60, *p* = 0.04) (Fig. 7).

## Discussion

The model of elastase-induced emphysema used herein induced cardiorespiratory changes that resembled the disease presentation observed in humans [20]. In this model, PSV compared to PCV: decreased Pmean,L, hyperinflation, mean linear intercept, and amphiregulin mRNA expression in the lung; reduced right ventricular afterload; and increased ultrastructural damage to the diaphragm without inducing changes in biomarkers associated with proteolysis. Our data suggest that PSV, while minimizing lung and cardiac impairment, may increase diaphragmatic damage as compared to PCV.

Our emphysema model induced increased areas of lung hyperinflation, mean linear intercept, amphiregulin mRNA expression in lung tissue, and MAFbx expression in the diaphragm, the latter suggesting diaphragmatic atrophy [21, 22]. Amphiregulin is known to be higher in emphysema patients who exhibit damaged epithelium [23]. Furthermore, upregulation of MAFbx mRNA expression has been found in the diaphragm of patients with emphysema, which is consistent with a reduction of diaphragm myosin content [24]. Pressure support and pressure-controlled ventilation were compared because both modes are frequently used in critically ill patients with emphysema [25]. Furthermore, a protective tidal volume (6 ml/kg) was applied to minimize its possible effects on lung injury. To the best of our knowledge, this is the first study to investigate the effects of PSV and PCV on cardiorespiratory function, lung histology, and biological markers associated with pulmonary inflammation, alveolar stretch, epithelial mechanotransduction, endothelial cell damage, extracellular matrix organization, and diaphragmatic injury in experimental emphysema.

### Effects of pressure support and pressure-controlled ventilation in control animals

We found that PSV compared to PCV, targeting a protective tidal volume, did not affect cardiorespiratory function, pulmonary morphology, or biomarkers of inflammation in the lung, but did increase MAFbx and MuRF1 in the diaphragm. However, in mechanically ventilated rabbits with healthy lungs, biphasic positive airway pressure associated with spontaneous breathing improved respiratory function and attenuated the activation of lung injury biological markers compared to PCV [26]. Differences between our data and those reported by Xia et al. may be attributed to the mode of assisted mechanical ventilation, since biphasic positive airway pressure, when associated with spontaneous breathing, may result in less stress and strain than pressure support [27], and to the use of low *V*_T_ in our study, which may have minimized the possible effects of ventilation mode on lung injury. In healthy rats, PSV compared to PCV has been shown to reduce histological damage [28] and protect against proteolysis [6] and decreased diaphragm protein synthesis [29], thus limiting diaphragmatic atrophy. In agreement with the literature, our data suggest that PSV minimized diaphragmatic injury in healthy animals.

### Effects of pressure support and pressure-controlled ventilation in emphysematous animals

Ti/Ttot was lower with PSV than with PCV, due to flow vs. time inspiratory–expiratory triggering [30]. As RR did not differ between PSV and PCV at 4 h of mechanical ventilation, the decrease in Ti/Ttot in PSV animals may be associated with a reduction in Ti and/or an increase in expiratory time (Te). Both the decrease in Ti, yielding reduced mean transpulmonary pressure, and the increase in Te, promoting lung exhalation, are likely to lead to less hyperinflation during PSV. In this context, Ti/Ttot correlated well with Pmean,L and hyperinflation. In fact, Ti/Ttot has been considered an important variable associated with dynamic hyperinflation in human patients with emphysema [31]. In addition, the increase in variability of Ti/Ttot [32] and RR may further improve lung homogeneity while reducing lung hyperinflation and mean linear intercept. The coefficient of variation of Ti/Ttot correlated well with Pmean,L and hyperinflation (Additional file 8: Figure S2), which also contributed towards decreased lung heterogeneity. It has been shown that one of addressable variable that tends to increase hyperinflation is the long Ti/Ttot [31]. Therefore, we may hypothesize that increased Ti/Ttot variability during mechanical ventilation can decrease hyperinflation.

PSV, as compared to PCV, increased PAT/PET and thus reduced right ventricular afterload, as demonstrated by the bell-shaped pulmonary artery flow waveform [12]. PAT/PET measured using echocardiography has been shown to correlate closely with invasively measured right ventricle systolic pressure [12]. This increase in PAT/PET may be explained by the lower transpulmonary pressure, regarded as the main determinant of pulmonary vascular resistance, by increased blood flow into the lung capillaries, which in turn reduces pulmonary vascular resistance secondary to vascular distension and recruitment [33], and by decreased right ventricular impedance as a result of reduced lung hyperinflation [34]. EF may appear not to differ between PSV and PCV because more time would be required for any changes to show; furthermore, visual detection of alterations in right ventricular area may be limited due to the lower sensitivity of EF compared to PAT/PET [35].

Even though PSV improved lung function and morphology, it had negative effects on the ultrastructure of the diaphragm, with increased fibrillar disarrangement, mitochondrial morphological abnormalities, and enlargement of the endoplasmic reticulum. These diaphragmatic changes may be attributed to variability in *V*_T_, respiratory rate, and Ti/Ttot inducing greater damage compared to a continuous effort. Moreover, we cannot rule out that total effort may also play a relevant role in the induction of muscle damage [36]. Therefore, it appears that an optimal work range during mechanical ventilation may exist [37]. Interestingly, unlike these early signs of ultrastructural diaphragm damage, the apparent absence of diaphragmatic biomarkers associated with muscle proteolysis during PSV may be associated with a delay in damage perception.

Amphiregulin mRNA expression in the lung was lower in PSV compared to PCV animals and correlated positively with Pmean,L and hyperinflation. In this context, amphiregulin has been reported to be associated with alveolar hyperinflation [38, 39]. Our data suggest that during PCV, emphysematous lungs are more susceptible to stretch (with increased amphiregulin expression) and to inflammation (with increased CINC-1 expression). It has been described that SP-D levels are lower in emphysema patients due to epithelial destruction of Clara cells and type-II alveolar cells responsible for SP-D production [40]. Additionally, SP-D deficiency induces emphysematous changes in murine lungs [41]. In the present study, SP-D increased similarly in both mechanical ventilation modes, which may suggest protective epithelial cell damage. Lysyl oxidase (LOX) catalyzes crosslinking of collagen and elastin, which play essential roles in maintaining the structural integrity of the lung extracellular matrix (ECM) [42]. In experimental emphysema, impairment of LOX production exacerbates alveolar destruction and emphysema [43]. In this line, both PCV and PSV reduced expression of LOXL1, a member of the LOX gene family, suggesting ECM impairment. Interestingly, these mechanical ventilation modes do not seem to affect endothelial cell damage or fibrogenesis, as no changes in VEGF, ANG-2, or PCIII mRNA expression were observed.

### Possible clinical implications

In healthy lungs, PSV may be helpful to maintain cardiorespiratory function while reducing diaphragmatic damage. On the other hand, in emphysema, even minor increases in inspiratory effort may promote diaphragmatic injury. Additionally, Ti/Ttot, its coefficient of variation, and RR must be controlled to maintain Pmean,L in a safe range so as to avoid ventilator-induced lung injury (VILI). Amphiregulin seems to be an important biological marker that could be used to monitor hyperinflation during mechanical ventilation in emphysema. Future clinical studies are warranted to better define the role of appropriate timing of PSV and level of inspiratory effort in emphysema.

### Limitations

This study has some limitations, which must be taken into account. First, no experimental model of emphysema is able to perfectly reproduce all features of human emphysema. However, the rat model of emphysema induced by multiple elastase instillations, as used in the present study, was associated with cardiorespiratory functional changes and diaphragmatic injury and may thus provide an efficient tool to better understand the role of PCV and PSV, with potential for translation into clinical practice. Second, our results cannot be extended to emphysema models with different degrees of severity. Third, a fixed PEEP level was applied to avoid introduction of a confounding factor. The PEEP level used in the current study (3 cmH_2_O), while often used in rats [20], was lower than the PEEP recommended for use in humans with emphysema (3–6 cmH_2_O), due to the dimensions of the lungs. We cannot rule out that different results might have been obtained with different PEEP levels. However, as emphysema model used herein was associated with pulmonary hypertension, high levels of PEEP should be avoided, as they can compress alveolar capillaries and elevate pulmonary vascular resistance regardless of ventilation mode. All animals were ventilated with the same PEEP level; therefore, differences in diaphragm injury could not be directlyrelated to PEEP. Fourth, mediators were measured in lung tissue, but not in blood.

## Conclusions

In the model of elastase-induced emphysema used herein, PSV, compared to PCV, reduced lung damage and improved cardiovascular function, but increased diaphragmatic injury. This is a first step towards understanding the effects of different modes of mechanical ventilation on VILI and ventilator-induced diaphragmatic damage in emphysema.

### Key messages

In experimental emphysema, pressure support vs. pressure-controlled ventilation decreased mean transpulmonary pressure, mean linear intercept, amphiregulin expression in the lung, and right ventricular afterload. Despite improving lung function and morphology, pressure support ventilation increased ultrastructural damage in the diaphragm.

## Additional files

Additional file 1: Figure S1. Tracings of airway pressure (Paw), esophageal pressure (Pes), flow, and volume over time during pressure-controlled (PCV) and pressure support ventilation (PSV) in control and emphysema animals. (DOCX 348 kb)
Additional file 2: Table S1. Target gene priners. (DOCX 14 kb)
Additional file 3: Table S2. Lung morphometry and expression of biological markers in lung and diaphragm tissues in the non-ventilated groups. (DOCX 13 kb)
Additional file 4: Table S3. Mean arterial pressure, arterial blood gases, and respiratory parameters at baseline (before randomization). (DOCX 15 kb)
Additional file 5: Table S4. Mean arterial pressure. (mmHg) (DOCX 13 kb)
Additional file 6: Table S5. Arterial blood gases. (DOCX 15 kb)
Additional file 7: Table S6. Gene expression of biological markers in lung tissue. (DOCX 13 kb)
Additional file 8: Figure S2. Spearman correlation between mean transpulmonary pressure (Pmean,L) and hyperinflation, with the coefficient of variation (CV) of ratio between inspiratory and total time (Ti/Ttot). The r value represents the correlation coefficient, and *p*, the respective *p* value. Statistical significance was accepted at *p* < 0.05. Black circles: Emphysema animals ventilated with PSV. White circles: Emphysema animals ventilated with PCV. (DOCX 82 kb)

## Abbreviations

36B4: Acidic ribosomal phosphoprotein P0
ANG-2: Angiopoietin-2
CINC-1: Cytokine-induced neutrophil chemoattractant
COPD: Chronic obstructive pulmonary disease
FIO_2_: Fraction of inspired oxygen
HR: Heart rate
I:E: Inspiratory/expiratory ratio
LOXL1: Lysyl oxidase-like 1
MAP: Mean arterial pressure
Mit: Mitochondria
NV: Non-ventilated
PaCO_2_: Arterial partial pressure of carbon dioxide
PaO_2_: Arterial partial pressure of oxygen
PAT: Pulmonary artery acceleration time
Paw: Airway pressure
PCIII: Type III procollagen
PCV: Pressure-controlled ventilation
PEEP: Positive end-expiratory pressure
PEEPi: Intrinsic positive end-expiratory pressure
Pes: Esophageal pressure
PET: Pulmonary artery ejection time
pHa: Arterial pH
Pmean,L: Mean transpulmonary pressure
Ppeak,L: Transpulmonary peak pressure
PSV: Pressure support ventilation
RR: Respiratory rate
RT-PCR: Real-time reverse transcription polymerase chain reaction
SP-D: Surfactant protein-D
VCAM-1: Vascular cell adhesion molecule 1
VEGF: Vascular endothelial growth factor
VILI: Ventilator-induced lung injury
*V*_T_: Tidal volume
Z: Z-disk
ZEEP: Zero end-expiratory pressure

## References

[1] GOLD GIfCOLD (2015). Global strategy for diagnosis, management and prevention of chronic obstructive pulmonary disease.

[2] Kawut SM, Poor HD, Parikh MA (2014). Cor pulmonale parvus in chronic obstructive pulmonary disease and emphysema: the MESA COPD study. J Am Coll Cardiol.

[3] Puig-Vilanova E, Aguilo R, Rodriguez-Fuster A (2014). Epigenetic mechanisms in respiratory muscle dysfunction of patients with chronic obstructive pulmonary disease. PLoS One.

[4] van Hees H, Ottenheijm C, Ennen L (2011). Proteasome inhibition improves diaphragm function in an animal model for COPD. Am J Physiol Lung Cell Mol Physiol.

[5] Chandra D, Wise RA, Kulkarni HS (2012). Optimizing the 6-min walk test as a measure of exercise capacity in COPD. Chest.

[6] Futier E, Constantin JM, Combaret L (2008). Pressure support ventilation attenuates ventilator-induced protein modifications in the diaphragm. Crit Care.

[7] Levine S, Nguyen T, Taylor N (2008). Rapid disuse atrophy of diaphragm fibers in mechanically ventilated humans. N Engl J Med.

[8] Demoule A, Jung B, Prodanovic H (2013). Diaphragm dysfunction on admission to the intensive care unit. Prevalence, risk factors, and prognostic impact-a prospective study. Am J Respir Crit Care Med.

[9] Saddy F, Sutherasan Y, Rocco PR (2014). Ventilator-associated lung injury during assisted mechanical ventilation. Semin Respir Crit Care Med.

[10] Hudson MB, Smuder AJ, Nelson WB (2012). Both high level pressure support ventilation and controlled mechanical ventilation induce diaphragm dysfunction and atrophy. Crit Care Med.

[11] Tejeda M, Boix JH, Alvarez F (1997). Comparison of pressure support ventilation and assist-control ventilation in the treatment of respiratory failure. Chest.

[12] Thibault HB, Kurtz B, Raher MJ (2010). Noninvasive assessment of murine pulmonary arterial pressure: validation and application to models of pulmonary hypertension. Circ Cardiovasc Imaging.

[13] Lang RM, Badano LP, Mor-Avi V (2015). Recommendations for cardiac chamber quantification by echocardiography in adults: an update from the American Society of Echocardiography and the European Association of Cardiovascular Imaging. J Am Soc Echocardiogr.

[14] Baydur A, Behrakis PK, Zin WA (1982). A simple method for assessing the validity of the esophageal balloon technique. Am Rev Respir Dis.

[15] Weibel ER (1990) Morphometry: stereological theory and practical methods. Models of lung disease-microscopy and structural methods Edited by Gil J Marcel Dekker. Academic Press, New York, p 199–247.

[16] Padilha GA, Henriques I, Lopes-Pacheco M (2015). Therapeutic effects of LASSBio-596 in an elastase-induced mouse model of emphysema. Front Physiol.

[17] Moraes L, Santos CL, Santos RS (2014). Effects of sigh during pressure control and pressure support ventilation in pulmonary and extrapulmonary mild acute lung injury. Crit Care.

[18] Akamine R, Yamamoto T, Watanabe M (2007). Usefulness of the 5′ region of the cDNA encoding acidic ribosomal phosphoprotein P0 conserved among rats, mice, and humans as a standard probe for gene expression analysis in different tissues and animal species. J Biochem Biophys Methods.

[19] Schmittgen TD, Livak KJ (2008). Analyzing real-time PCR data by the comparative C(T) method. Nat Protoc.

[20] Henriques I, Padilha GA, Huhle R (2016). Comparison between variable and conventional volume-controlled ventilation on cardiorespiratory parameters in experimental emphysema. Front Physiol.

[21] Lecker SH, Jagoe RT, Gilbert A (2004). Multiple types of skeletal muscle atrophy involve a common program of changes in gene expression. FASEB J.

[22] McKinnell IW, Rudnicki MA (2004). Molecular mechanisms of muscle atrophy. Cell.

[23] de Boer WI, Hau CM, van Schadewijk A (2006). Expression of epidermal growth factors and their receptors in the bronchial epithelium of subjects with chronic obstructive pulmonary disease. Am J Clin Pathol.

[24] Testelmans D, Crul T, Maes K (2010). Atrophy and hypertrophy signalling in the diaphragm of patients with COPD. Eur Respir J.

[25] Esteban A, Frutos-Vivar F, Muriel A (2013). Evolution of mortality over time in patients receiving mechanical ventilation. Am J Respir Crit Care Med.

[26] Xia J, Sun B, He H (2011). Effect of spontaneous breathing on ventilator-induced lung injury in mechanically ventilated healthy rabbits: a randomized, controlled, experimental study. Crit Care.

[27] Gama de Abreu M, Cuevas M, Spieth PM (2010). Regional lung aeration and ventilation during pressure support and biphasic positive airway pressure ventilation in experimental lung injury. Crit Care.

[28] Oliveira Ade S, Costa LB, Assis Tde O (2012). Effects of controlled and pressure support mechanical ventilation on rat diaphragm muscle. Acta Cir Bras.

[29] Hudson MB, Smuder AJ, Nelson WB (2015). Partial support ventilation and mitochondrial-targeted antioxidants protect against ventilator-induced decreases in diaphragm muscle protein synthesis. PLoS One.

[30] Henzler D, Pelosi P, Bensberg R (2006). Effects of partial ventilatory support modalities on respiratory function in severe hypoxemic lung injury. Crit Care Med.

[31] Marini JJ (2011). Dynamic hyperinflation and auto-positive end-expiratory pressure: lessons learned over 30 years. Am J Respir Crit Care Med.

[32] Di Mussi R, Spadaro S, Mirabella L (2016). Impact of prolonged assisted ventilation on diaphragmatic efficiency: NAVA versus PSV. Crit Care.

[33] Cherpanath TG, Lagrand WK, Schultz MJ (2013). Cardiopulmonary interactions during mechanical ventilation in critically ill patients. Neth Hear J.

[34] Ranieri VM, Grasso S, Fiore T (1996). Auto-positive end-expiratory pressure and dynamic hyperinflation. Clin Chest Med.

[35] Mazurek JA, Forfia PR (2013). Enhancing the accuracy of echocardiography in the diagnosis of pulmonary arterial hypertension: looking at the heart to learn about the lungs. Curr Opin Pulm Med.

[36] Vassilakopoulos T, Petrof BJ (2004). Ventilator-induced diaphragmatic dysfunction. Am J Respir Crit Care Med.

[37] Goligher EC, Fan E, Herridge MS (2015). Evolution of diaphragm thickness during mechanical ventilation. Impact of inspiratory effort. Am J Respir Crit Care Med.

[38] Dolinay T, Kaminski N, Felgendreher M (2006). Gene expression profiling of target genes in ventilator-induced lung injury. Physiol Genomics.

[39] Samary CS, Santos RS, Santos CL (2015). Biological impact of transpulmonary driving pressure in experimental acute respiratory distress syndrome. Anesthesiology.

[40] Sims MW, Tal-Singer RM, Kierstein S (2008). Chronic obstructive pulmonary disease and inhaled steroids alter surfactant protein D (SP-D) levels: a cross-sectional study. Respir Res.

[41] Botas C, Poulain F, Akiyama J (1998). Altered surfactant homeostasis and alveolar type II cell morphology in mice lacking surfactant protein D. Proc Natl Acad Sci U S A.

[42] Mizuno S, Yasuo M, Bogaard HJ (2011). Inhibition of histone deacetylase causes emphysema. Am J Physiol Lung Cell Mol Physiol.

[43] Warburton D, Gauldie J, Bellusci S (2006). Lung development and susceptibility to chronic obstructive pulmonary disease. Proc Am Thorac Soc.

