# Supplementary material for: Comparison between effects of pressure support and pressure-controlled ventilation on lung and diaphragmatic damage in experimental emphysema
Source: Intensive Care Med Exp. 2016 Oct 19;4:35. doi: 10.1186/s40635-016-0107-0 (PMC5071308; doi:10.1186/s40635-016-0107-0)
Supplement: Additional file 1: Figure S1. — Tracings of airway pressure (Paw), esophageal pressure (Pes), flow, and volume over time during pressure-controlled (PCV) and pressure support ventilation (PSV) in control and emphysema animals. (DOCX 348 kb) [file 40635_2016_107_MOESM1_ESM.docx]

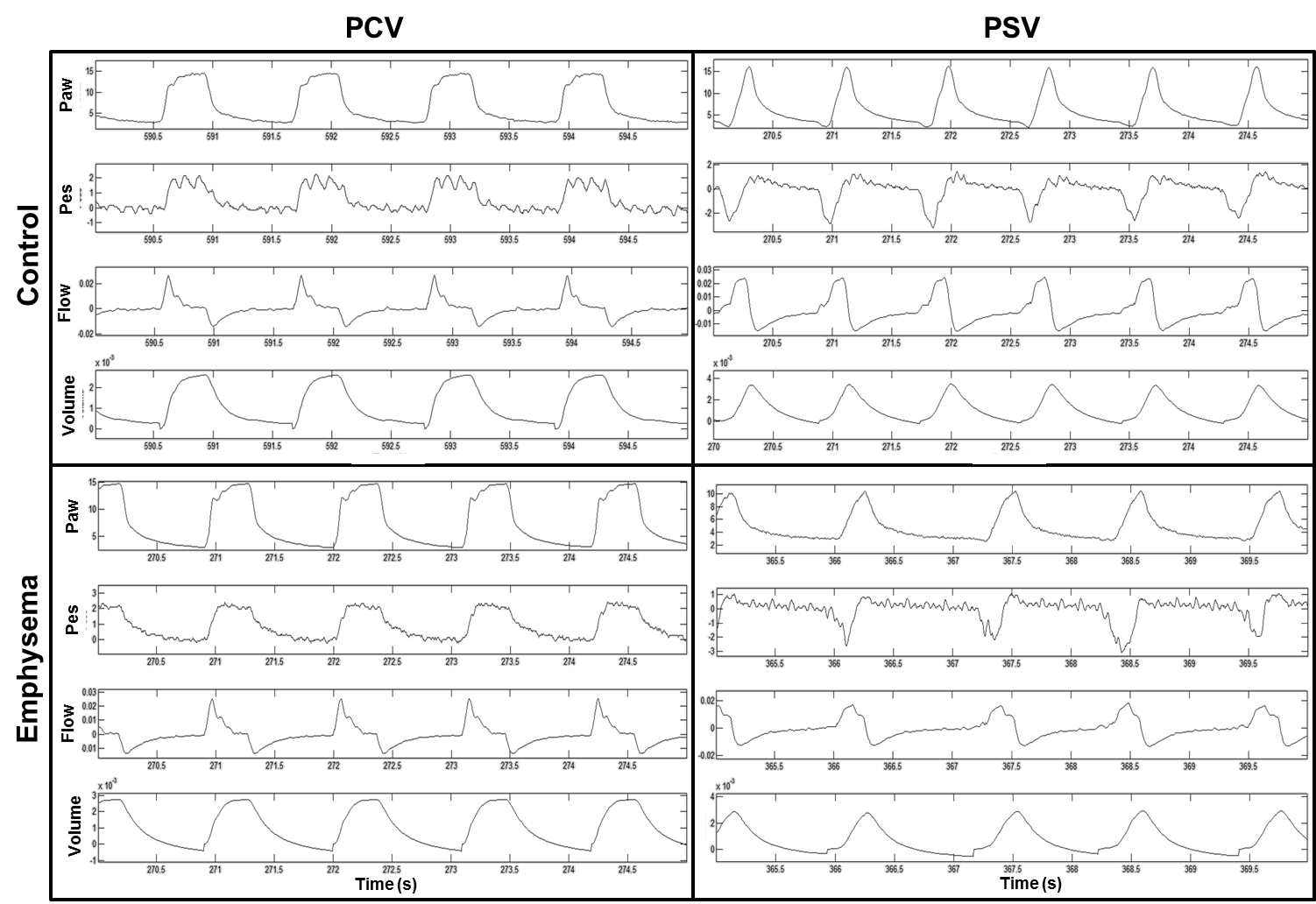


Figure 1S. Tracings of airway pressure (Paw), esophageal pressure (Pes), flow, and volume over time during pressure-controlled (PCV) and pressure-support ventilation (PSV) in control and emphysema animals.
