# Supplementary material for: Comparison between effects of pressure support and pressure-controlled ventilation on lung and diaphragmatic damage in experimental emphysema
Source: Intensive Care Med Exp. 2016 Oct 19;4:35. doi: 10.1186/s40635-016-0107-0 (PMC5071308; doi:10.1186/s40635-016-0107-0)
Supplement: Additional file 2: Table S1. — Target gene priners. (DOCX 14 kb) [file 40635_2016_107_MOESM2_ESM.docx]

| **Table 1S. Target gene priners** |  | |
| --- | --- | --- |
| **Gene** | **Forward** | **Reverse** |
| *Lungs* |  |  |
| CINC-1 | 5´-TGC ACC CAA ACC GAA GTC AT-3´ | 5´-TTG TCA GAA GCC AGC GTT CAC-3´ |
| Amphiregulin | 5´-TTTCGCTGGCGCTCTCA-3´ | 5´-TTCCAACCCAGCTGCATAATG-3´ |
| SP-D | 5´-AAATCTTCAGGGCGGCAAA-3´ | 5´-GGCCTGCCTGCACATCTC-3´ |
| LOXL1 | 5´-CTG TAT TCC TTG CGT TGT GC-3´ | 5´-TGG TGA CAG CTA TGC CAC TC-3´ |
| VEGF | 5´-CAG AAA GCC CAT GAA GTG GT-3´ | 5´-ACA CAG GAC GGC TTG AAG AT-3´ |
| ANG-2 | 5´-CAGCCAACCAGGTGATT-3´ | 5´-AAGTTGGAAGGACCACATGC-3´ |
| PCIII | 5´-ACC TGG ACC ACA AGG ACA C-3´ | 5´-TGG ACC CAT TTC ACC TTT C-3´ |
| VCAM-1 | 5´-TGCACGGTCCCTAATGTGTA-3´ | 5′-TGCCAATTTCCTCCCTTAAA-3′ |
| *Diaphragm* |  |  |
| MAFbx | 5´-TCA CAG CTC ACA TCC CTG AG-3´ | 5´-GAC TTG CCG ACT CTC TGG AC-3´ |
| MuRF-1 | 5´-TCA CAG CTC ACA TCC CTG AG-3´ | 5´-GAC TTG CCG ACT CTC TGG AC-3´ |
| 36B4 | 5´-GGATCACTCAGGAGCAGGAG-3´ | 5´-CTTGGCACTCAAGAGGAAGG-3´ |

CINC-1, cytokine-induced neutrophil chemoattractant-1; SP-D, surfactant protein D; LOXL1, lysyl oxidase like 1; VEGF, vascular endothelial growth factor; ANG-2, angiopoietin-2; PCIII, procollagen type III; VCAM-1, vascular cell adhesion molecule; MAFbx, muscle atrophy F-Box; MuRF1, muscle RING Finger-1.
