# Supplementary material for: Comparison between effects of pressure support and pressure-controlled ventilation on lung and diaphragmatic damage in experimental emphysema
Source: Intensive Care Med Exp. 2016 Oct 19;4:35. doi: 10.1186/s40635-016-0107-0 (PMC5071308; doi:10.1186/s40635-016-0107-0)
Supplement: Additional file 3: Table S2. — Lung morphometry and expression of biological markers in lung and diaphragm tissues in the non-ventilated groups. (DOCX 13 kb) [file 40635_2016_107_MOESM3_ESM.docx]

**Table 2S. Lung morphometry and expression of biological markers in lung and diaphragm tissues in the non-ventilated groups**

| **Group** | **Lm**  **(μm)** | **Hyperinflation**  **(%)** | **Lungs**  **Amphiregulin**  **(fold changes relative to control)** | **Diaphragm**  **MAFBx**  **(fold changes relative to control)** |
| --- | --- | --- | --- | --- |
| **Control** | 46.3 (41.4-51.8) | 0 (0-0) | 1.3 (0.5-2.1) | 1.1 (0.6-1.4) |
| **Emphysema** | 65.7 (63.0-72.8) ** | 42 (33.7-43.4) ** | 3.7 (3.2-4.3)** | 2.0 (1.5-2.5)** |

Lm: mean linear intercept. Real-time polymerase chain reaction analysis of amphiregulin and muscle atrophy F-Box (MAFbx). Relative gene expression was calculated as a ratio of the average gene expression levels compared with the reference gene (*36B4*) and expressed as fold change relative to control non-ventilated animals. Values are medians (interquartile range) of 6 animals in each group. ** Significantly different from control non-ventilated animals (p<0.05)
