# Supplementary material for: Comparison between effects of pressure support and pressure-controlled ventilation on lung and diaphragmatic damage in experimental emphysema
Source: Intensive Care Med Exp. 2016 Oct 19;4:35. doi: 10.1186/s40635-016-0107-0 (PMC5071308; doi:10.1186/s40635-016-0107-0)
Supplement: Additional file 4: Table S3. — Mean arterial pressure, arterial blood gases, and respiratory parameters at baseline (before randomization). (DOCX 15 kb) [file 40635_2016_107_MOESM4_ESM.docx]

|  | **Control** | | **Emphysema** | |
| --- | --- | --- | --- | --- |
|  | **PCV** | **PSV** | **PCV** | **PSV** |
|  |  |  |  |  |
| **MAP (mmHg)** | 147.7 ± 9.2 | 142.3 ± 17.9 | 138.6 ± 35.7 | 148.2 ± 22.7 |
| **Arterial blood gases** |  |  |  |  |
| pH_a_ | 7.34 ± 0.03 | 7.37 ± 0.05 | 7.35 ± 0.06 | 7.38 ± 0.05 |
| PaO_2_ (mmHg) | 89.9 ± 14.7 | 82.2 ± 20.3 | 111.4 ± 39.9 | 79.7 ± 14.8 |
| PaCO_2_ (mmHg) | 41.4 ± 4.2 | 44.2 ± 6.5 | 44.9 ± 8.6 | 43.7 ± 8.4 |
| Bicarbonate (mmol.L^-1^) | 22.5 ± 3.5 | 25.1 ± 2.3 | 24.6 ± 3.9 | 25.8 ± 3.7 |
| **Respiratory parameters** |  |  |  |  |
| V_T_ (mL.kg^-1^) | 5.4 ± 0.3 | 5.4 ± 0.6 | 5.3 ± 0.5 | 5.4 ± 0.5 |
| CV of V_T_ (%) | 3.1 ± 2.0 | 3.9 ± 0.0 | 6.5 ± 3.0 | 5.5 ± 3.7 |
| RR (breaths.min^-1^) | 47.1 ± 7.3 | 45.0 ± 5.5 | 59.0 ± 16.7 | 61.0 ± 12.8 |
| CV of RR (%) | 23.1 ± 0.2 | 15.6 ± 0.1 | 27.3 ± 0.1 | 22.2 ± 0.2 |
| Ti/Ttot (s) | 0.22 ± 0.06 | 0.23 ± 0.04 | 0.32 ± 0.10 | 0.31 ± 0.10 |
| CV of Ti/Ttot (%) | 15.0 ± 2.9 | 19.4 ± 5.4 | 24.2 ± 13.0 | 18.7 ± 11.9 |
| Ppeak,L (cmH_2_O) | 12.4 ± 0.8 | 11.2 ± 1.5 | 10.3 ± 0.4 | 11.8 ± 1.5 |
| Pmean,L (cmH_2_O) | 2.1 ± 0.3 | 1.8 ± 0.3 | 2.3 ± 0.7 | 2.7 ± 0.6 |
| PTP/min (cmH_2_O.s.min^-1^) | 6.6 ± 3.7 | 6.3 ± 4.5 | 17.9 ± 13.7 | 26.3 ± 24.7 |
| P_0.1_ (cmH_2_O) | -0.8 ± 0.6 | -0.9 ± 0.7 | -0.6 ± 0.5 | -1.9 ± 1.1 |

**Table 3S. Mean arterial pressure, arterial blood gases, and respiratory parameters at baseline (before randomization).**

All data were obtained at the Baseline time point, before randomization. PCV, pressure-controlled ventilation; PSV, pressure support ventilation; MAP, mean arterial pressure; pH_a_, arterial pH; PaO_2_, partial pressure of arterial oxygen; PaCO_2_: partial pressure of arterial carbon dioxide; V_T_, tidal volume; CV of V_T_, coefficient of variation of tidal volume; RR, respiratory rate; CV of RR, coefficient of variation of respiratory rate; Ti/Ttot, inspiratory time divided by total respiratory cycle time; Ppeak,L, transpulmonary peak pressure; Pmean,L, transpulmonary mean pressure; PTP/min, pressure–time product per minute; P_0.1_, esophageal pressure generated 100 ms after onset of inspiratory effort. Values are means ± SD of 6 animals.
