# Supplementary material for: Comparison between effects of pressure support and pressure-controlled ventilation on lung and diaphragmatic damage in experimental emphysema
Source: Intensive Care Med Exp. 2016 Oct 19;4:35. doi: 10.1186/s40635-016-0107-0 (PMC5071308; doi:10.1186/s40635-016-0107-0)
Supplement: Additional file 5: Table S4. — Mean arterial pressure. (mmHg) (DOCX 13 kb) [file 40635_2016_107_MOESM5_ESM.docx]

| **Group** | | **T0** | **T2** | **T4** |
| --- | --- | --- | --- | --- |
| **Control** | **PCV** | 118.7 ± 28.2 | 105.5 ± 11.5 | 83.0 ± 17.8 |
|  | **PSV** | 126.7 ± 24.8 | 108.0 ± 31.0 | 100.2 ± 33.0 |
| **Emphysema** | **PCV** | 130.1 ± 19.0 | 109.2 ± 14.0 | 96.6 ± 19.3 |
|  | **PSV** | 132.8 ± 22.8 | 112.8 ± 8.1 | 90.5 ± 27.3 |

**Table 4S. Mean arterial pressure (mmHg)**

PCV, pressure-controlled ventilation; PSV, pressure support ventilation. T0: immediately after randomization; T2 and T4: 2 and 4 hours of mechanical ventilation after randomization, respectively. Values are means ± SD of 6 animals at each time point.
