# Supplementary material for: Comparison between effects of pressure support and pressure-controlled ventilation on lung and diaphragmatic damage in experimental emphysema
Source: Intensive Care Med Exp. 2016 Oct 19;4:35. doi: 10.1186/s40635-016-0107-0 (PMC5071308; doi:10.1186/s40635-016-0107-0)
Supplement: Additional file 6: Table S5. — Arterial blood gases. (DOCX 15 kb) [file 40635_2016_107_MOESM6_ESM.docx]

| **Parameter** | **Group** | | **T0** | **T2** | **T4** | **Time effect** | **Group effect** | **Interaction** |
| --- | --- | --- | --- | --- | --- | --- | --- | --- |
| **pH_a_** | **Control** | **PCV** | 7.36 ± 0.07 | 7.35 ± 0.04 | 7.33 ± 0.05 | ns | ns | ns |
|  |  | **PSV** | 7.34 ± 0.03 | 7.34 ± 0.06 | 7.32 ± 0.10 |  |  |  |
|  | **Emphysema** | **PCV** | 7.35 ± 0.04 | 7.32 ± 0.09 | 7.34 ± 0.03 |  |  |  |
|  |  | **PSV** | 7.34 ± 0.03 | 7.38 ± 0.04 | 7.33 ± 0.09 |  |  |  |
| **PaO_2_**  **(mmHg)** | **Control** | **PCV** | 115.6 ± 34.5 | 153.7 ± 14.7 | 130.3 ± 32.0 | p<0.001 | ns | ns |
|  |  | **PSV** | 115.0 ± 9.7 | 157.0 ± 33.2 | 150.3 ± 28.1 |  |  |  |
|  | **Emphysema** | **PCV** | 119.2 ± 28.7 | 157.6 ± 18.0 | 139.0 ± 25.0 |  |  |  |
|  |  | **PSV** | 127.7 ± 21.7 | 164.9 ± 15.6 | 140.7 ± 23.8 |  |  |  |
| **PaCO_2_**  **(mmHg)** | **Control** | **PCV** | 37.1 ± 5.0 | 42.9 ± 8.1 | 42.9 ± 7.4 | ns | ns | ns |
|  |  | **PSV** | 42.2 ± 9.0 | 44.3 ± 10.9 | 49.0 ± 17.8 |  |  |  |
|  | **Emphysema** | **PCV** | 44.9 ± 4.8 | 41.5 ± 7.2 | 41.7 ± 2.9 |  |  |  |
|  |  | **PSV** | 48.0 ± 6.8 | 39.1 ± 6.9 | 46.9 ± 15.8 |  |  |  |
| **Bicarbonate**  **(mmol.L^-1^)** | **Control** | **PCV** | 20.8 ± 2.0 | 22.7 ± 2.3 | 21.9 ± 2.1 | p<0.01 | ns | p<0.05 |
|  |  | **PSV** | 22.7 ± 5.4 | 22.4 ± 3.5 | 23.5 ± 4.0 |  |  |  |
|  | **Emphysema** | **PCV** | 24.7 ± 2.2 | 20.5 ± 3.0 | 21.7 ± 2.4 |  |  |  |
|  |  | **PSV** | 25.4 ± 2.1 | 22.1 ± 2.2 | 23.0 ± 2.7 |  |  |  |

**Table 5S. Arterial blood gases**

PCV, pressure-controlled ventilation; PSV, pressure support ventilation; pH_a_, arterial pH; PaO_2_, partial pressure of arterial oxygen; PaCO_2_, partial pressure of arterial carbon dioxide. T0: immediately after randomization; T2 and T4: 2 and 4 hours of mechanical ventilation after randomization, respectively. Values are means ± SD of 6 animals at each time point.
