# Supplementary material for: Comparison between effects of pressure support and pressure-controlled ventilation on lung and diaphragmatic damage in experimental emphysema
Source: Intensive Care Med Exp. 2016 Oct 19;4:35. doi: 10.1186/s40635-016-0107-0 (PMC5071308; doi:10.1186/s40635-016-0107-0)
Supplement: Additional file 7: Table S6. — Gene expression of biological markers in lung tissue. (DOCX 13 kb) [file 40635_2016_107_MOESM7_ESM.docx]

| **Group** | | **VEGF** | | **ANG-2** | **PCIII** | **VCAM-1** | |
| --- | --- | --- | --- | --- | --- | --- | --- |
| **Control** | **NV** | 0.8 (0.6-1.7) | 0.7 (0.4-3.0) | | 5.8 (0.0-40.0) | | 1.2 (0.6-1.4) |
|  | **PCV** | 3.1 (2.0-6.4) | 1.0 (0.9-1.6) | | 3.4 (1.0-5.4) | | 1.4 (0.7-3.5) |
|  | **PSV** | 2.4 (1.9-3.2) | 0.7 (0.3-0.8) | | 3.4 (2.1-41.4) | | 1.8 (0.9-2.9) |
| **Emphysema** | **NV** | 1.0 (0.5-1.2) | | 1.1 (0.6-1.7) | 3.5 (0.1-18.9) | 1.7 (0.4-2.2) | |
|  | **PCV** | 0.8 (0.4-1.7) | | 0.9 (0.7-1.8) | 5.6 (2.0-9.6) | 1.0 (0.3-5.9) | |
|  | **PSV** | 0.8 (0.4-1.7) | | 0.7 (0.3-1.0) | 5.9 (3.7-9.5) | 6.4 (0.8-6.5) | |

**Table 6S. Gene expression of biological markers in lung tissue**

Real-time polymerase chain reaction analysis of vascular endothelial growth factor (VEGF), angiopoietin (ANG)-2, type III procollagen (PCIII), and vascular cell adhesion molecule (VCAM)-1. Relative gene expression was calculated as a ratio of the average gene expression levels compared with the reference gene (*36B4*) and expressed as fold change relative to NV (non-ventilated) animals in the Control and Emphysema groups. PCV, pressure-controlled ventilation; PSV, pressure support ventilation. Values are medians (interquartile range) of 6 animals in each group.
