# Supplementary material for: Comparison between effects of pressure support and pressure-controlled ventilation on lung and diaphragmatic damage in experimental emphysema
Source: Intensive Care Med Exp. 2016 Oct 19;4:35. doi: 10.1186/s40635-016-0107-0 (PMC5071308; doi:10.1186/s40635-016-0107-0)
Supplement: Additional file 8: Figure S2. — Spearman correlation between mean transpulmonary pressure (Pmean,L) and hyperinflation, with the coefficient of variation (CV) of ratio between inspiratory and total time (Ti/Ttot). The r value represents the correlation coefficient, and p, the respective p value. Statistical significance was accepted at p < 0.05. Black circles: Emphysema animals ventilated with PSV. White circles: Emphysema animals ventilated with PCV. (DOCX 82 kb) [file 40635_2016_107_MOESM8_ESM.docx]

Figure 2S. Spearman correlation between mean transpulmonary pressure (Pmean,L) and hyperinflation, with the coefficient of variation (CV) of ratio between inspiratory and total time (Ti/Ttot). The r value represents the correlation coefficient, and p, the respective *p*-value. Statistical significance was accepted at *p* < 0.05. **Black circles**: Emphysema animals ventilated with PSV. **White circles:** Emphysema animals ventilated with PCV.
